# Is Sustainable Consumption a Sufficient Motivator for Consumers to Adopt Meat Alternatives? A Consumer Perspective on Plant-Based, Cell-Culture-Derived, and Insect-Based Alternatives

**DOI:** 10.3390/foods13111627

**Published:** 2024-05-23

**Authors:** Nayyer Rehman, Victoria Edkins, Nives Ogrinc

**Affiliations:** 1 WRG Europe Ltd., 26–28 Southernhay East, Exeter EX1 1NS, UK; nayyer@wrgeurope.co.uk (N.R.); victoria.edkins@brcch.org (V.E.); 2 Jožef Stefan International Postgraduate School, Jamova 39, 1000 Ljubljana, Slovenia; 3 Department of Environmental Sciences, Jožef Stefan Institute, Jamova 39, 1000 Ljubljana, Slovenia

**Keywords:** consumer perception, consumer acceptance, alternative proteins, cell-culture-derived meat, edible insects, plant-based meat

## Abstract

This study investigates consumer preference and acceptance of three meat alternatives—plant-based, lab-grown, and insect-based—as sustainable choices to meet the demands of a growing population and evolving food systems. Insights were gathered from European consumers regarding their perceptions and consumption patterns using a mixed-methods approach. The approach employed a questionnaire followed by focus group discussions conducted in Slovenia and the UK to understand the motivations and barriers behind their responses. The UK and Slovenia were chosen as they provided the highest response rates to the questionnaire and they have differing legislation. The results show that plant-based alternatives are the most familiar and accepted option, while lab-grown meat and insect-based products are less familiar and have lower acceptance rates. Moreover, they show that although sustainability factors are important to consumers, they are not their only concern; health and nutrition are the primary motivators for choosing meat alternatives. These are followed closely by sensory appeal, pricing, and a preference for natural, minimally processed options. Based on insights from the focus groups, strategies to overcome the barriers to the acceptance of meat alternatives should include targeted product categorisation and placement, educational campaigns, effective use of media, and greater transparency in product information.

## 1. Introduction

With the projected global population set to reach 10 billion by 2050, it is crucial to increase food production by 70% to meet the growing demand, particularly for protein [1,2]. A solution to this challenge has emerged in the form of protein sources derived from plants, microorganisms, insects, or cultivated in laboratories. These food sources, often referred to as ‘alternative protein sources to meat’, ‘alternative proteins’, or ‘meat alternatives’, have gained attention as a promising solution to address the challenges posed by the rapidly expanding global population [3,4,5].

Alternative protein sources, such as insects, are recognised as sustainable solutions to the environmental issues associated with high meat consumption [6,7,8]. Insect production has been found to require significantly less land, using only 0.36 to 3.6 m^2^ per kg of biomass compared to 23.1 m^2^ for beef, 4.64 m^2^ for chicken, and 1.48 m^2^ for standard feed. Additionally, it has a lower carbon footprint, with emissions averaging just 0.3 to 3.0 kg of CO_2_ equivalents per kg of biomass [9].

Insects also show more efficient feed conversion ratios (FCRs) than traditional livestock, meaning they can convert feed into edible meat more effectively, using fewer resources for the same amount of protein. For instance, the FCRs of the black soldier fly (1.4 to 2.6) and the yellow mealworm (3.8 to 6.1) are much more efficient than for cows (8.8) [10].

Frass, the primary by-product of insect farming, is an effective organic fertiliser that can enhance soil fertility. Using frass could reduce reliance on chemical fertilisers, which are linked to water pollution and soil acidification, and contribute to a minimal waste and emissions profile of insect farming [11]. These benefits make insects a viable and sustainable alternative to conventional meat production, requiring less land and water and exhibiting rapid growth rates, making them an appealing option for sustainable agriculture [12,13].

The environmentally friendly aspect of cell-cultured-derived meat (herein referred to as CCDM) has been a topic of debate due to its production process. CCDM is developed in a laboratory by harvesting animal cells and cultivating them in a controlled environment [14], which requires significant energy. However, a proper life cycle assessment of CCDM has not yet been conducted due to the lack of information about the processes and materials involved, complicating the understanding of its environmental impacts. Despite the current limitations, CCDM is suggested to have the potential to be more environmentally friendly than conventional meat products, especially if sustainable energy sources are employed [15]. For instance, using renewable energy during production could lower the impact of greenhouse gas emissions compared to beef production. Currently, these emissions are expected to be similar to those of conventional poultry farms and, in some cases, higher than those of efficient poultry systems [16].

The land use for producing CCDM varies based on the feedstock sources that provide nutrients to the cells. Using efficient systems, such as those involving blue-green algae, can make CCDM require less land than beef and chicken [17]. However, the benefits are less predictable when traditional feeds such as soya and maize are used [18]. The biggest reason for CCDM gaining traction is as a humane alternative to slaughtering whole animals, thereby promoting animal welfare and establishing ethical alternatives for consuming traditional meat products [19].

Plant-based alternatives can also positively impact the environment and animal welfare. For instance, Poore and Namecek [20] found that peas are a more efficient protein source to grow, requiring less land (1.2–6.4 m^2^ per 100 g of protein) in comparison to beef (42–369 m^2^ per 100 g of protein) and poultry (3.8–9.2 m^2^ per 100 g of protein) production.

The European Food Safety Authority (EFSA) concluded that the median mean meat consumption for adults in over 20 EU member states was 35 g/day. At the 95th percentile, meat consumption varied from 20 g/day (21% of Swedish consumers) to 237 g/day (88% of Austrian consumers) [21]. This widespread consumption raises concerns for individuals who regularly consume red meat as it has been associated with chronic health conditions such as type 2 diabetes, cardiovascular disease, colorectal cancer, and other forms of cancer, which in some cases may lead to mortality. Conversely, alternative proteins have been found to provide improved nutritional quality and lower levels of saturated fats and cholesterol, thus offering significant health benefits [22,23]. According to a 2019 global survey, 40% of consumers are trying to reduce their intake of animal proteins, and 10% avoid red meat altogether. With an increasing number of people looking to cut down on meat and embrace vegetarian or vegan lifestyles, meat alternatives offer a practical solution to cater to different dietary preferences [24].

Although existing research on the acceptance of alternative proteins yields substantial data on consumer perspectives, willingness to adopt these products, and the influencing factors such as taste, texture, price, health benefits, environmental sustainability, and the impact of social and cultural influences on consumption habits [3,25,26,27,28,29,30,31,32,33,34], the market share of meat substitutes remain low [35,36]. To better understand this reluctance to adopt alternative proteins, it requires a deeper understanding of consumer perceptions and attitudes, and how these factor into their evaluation of the benefits or potential risks of switching to alternative proteins or reducing their traditional meat consumption, and thus their role in their decision to include these products in the diet [37,38,39].

Siegrist et al. [40] suggest engaging directly with consumers and actively seeking their input to design campaigns and develop strategies based on consumer preferences and concerns, to address the challenge of consumer acceptance. This approach not only assists in identifying specific consumer groups and developing tailored strategies for them [41,42] but it also raises the intriguing question of how people’s opinions might differ in regions with similar geographical locations but differing legal frameworks.

In the European Union (EU), four insect species (yellow mealworm, migratory locust, house cricket, and lesser mealworm) are currently classified as novel foods under Regulation (EU) 2015/2283 [43,44]. As a result of this classification, insect-based foods have become increasingly common in many EU countries, resulting in sales of around 500 tonnes in 2019, and are expected to reach approximately 260,000 tonnes by 2030 [45]. However, the situation differs in the UK, where regulations for novel foods have had to be re-evaluated in the post-Brexit era, leading to adverse effects on manufacturers of insect-based products [46]. These regulatory adjustments have impacted the availability of such foods in supermarkets, potentially rendering them unfamiliar to consumers in this country.

On the other hand, CCDM has also been the subject of debate regarding its production process and has had varying official statements in the UK and different regulations within the EU. For example, while it has been entirely banned in Italy [47], CCDM burgers are already scheduled for government-approved consumer tasting trials in the Netherlands [48].

In contrast, plant-based alternatives have encountered minimal production and safety concerns in Europe, where the plant-based food market is expected to grow at a CAGR of 10.1% from 2022 to 2029 [49]. Their primary challenge lies in labelling restrictions, such as the guidelines laid out in Regulation (EU) 1169/2011 regarding accurate labelling of ingredients and whether the product is of animal or plant origin [50]. As a result, plant-based manufacturers in France, for example, have been prohibited from using terms like “sausage” and “steak” for their products [51].

Despite numerous studies that examined consumer perceptions of alternative proteins and provided valuable insights for intervention strategies [39], consumers continue to reject these options, a sentiment reflected in their purchasing behaviour [52,53,54,55,56]. In addition, the existing literature provides limited insight into the factors that influence the acceptance of different alternative protein sources when compared to one another. This study addresses precisely that by placing consumers at the centre of the discussion of these barriers to consumption, engaging them to develop strategies that will yield results that prioritise consumer inputs, and assessing key differences in how consumers view acceptance of different meat alternatives.

The aim of this study is threefold: to assess current consumer perceptions and motivations towards alternative protein sources to meat and, subsequently, to evaluate whether sustainability-related factors alone motivate consumers to switch to these meat alternatives; then, finally, to understand how the source of alternative proteins can affect consumer perceptions, motivations, and barriers. With those purposes, this study selected three different types of alternative proteins, each originating from a different source: plant-based foods, edible insects and insect-based foods, and CCDM.

Additionally, this study examines the barriers preventing consumers from choosing alternative protein sources, and provides practical recommendations to address these barriers based on direct consumer insights. It must be considered that the availability of products and, by extension, consumer perception of these products in a country are highly dependent on its regulations [57]. For a meaningful comparison, two European countries with the highest response rates and differing regulatory environments were selected to understand the differences in opinions that can arise due to geographical location: Slovenia, falling under the EU jurisdiction, and the UK, which does not.

By employing both qualitative and quantitative approaches, we aim to enhance our understanding and corroborate our findings on consumer perceptions, motivations, and barriers, which will assist food companies, retailers, and policymakers in identifying specific population segments more inclined to try alternative meat products and those who are not, enabling them to tailor communication and marketing efforts and bridge the gap between consumer awareness and acceptance of alternative protein sources to meat.

## 2. Materials and Methods

This study employed a two-stage, mixed-methods approach to address the identified research gaps, including a questionnaire and focus groups. Participants were provided with clear guidelines regarding data processing and confidentiality in accordance with the GDPR guidelines [58]. Since this study required voluntary participation, participants were requested to consent before attempting the questionnaire or participating in the focus group discussions. No personal information was collected, and all ethical practices were rigorously followed to ensure anonymity. This study also strictly adhered to the principles outlined in the European Commission’s Ethics for Researchers [59] and the Social Research Association (SRA)’s Ethical Guidelines for Social Research [60].

### 2.1. Questionnaire Design and Data Collection and Analysis

The questionnaire (Supplementary Materials), designed to identify factors influencing consumer perception and purchasing behaviour of alternative protein sources to meat in Europe, was created on the Typeform survey platform. The questionnaire began by communicating privacy and data protection guidelines to participants, followed by questions on demographic information, including age, country of residence, sex, and education level.

The questionnaire’s core components were derived from the ‘Alternative Proteins: Consumer Survey’ report [61] and adapted to meet this study’s requirements, incorporating insights from experts within the institute specialising in this field. The questionnaire was structured in four sections. The first section focused on identifying the key factors influencing grocery purchasing decisions, aiming to gather insights into the drivers behind consumers’ choices. In the second section, respondents were asked why they believed alternative protein sources had been introduced to the market, assessing their existing perceptions of these foods, while section three aimed to measure respondent awareness of various alternative protein options available in the market. The final section consisted of eight questions exploring safety perceptions and consumption habits for plant-based alternatives, edible insects, and CCDM. It is important to note that CCDM is still in the experimental development phase in European countries; thus, it is not yet available to supermarket consumers. The questions for this category were adapted accordingly.

Before the virtual distribution of the questionnaire, two test sessions were conducted with colleagues to ensure the questionnaire’s accessibility for collecting online responses. After that, the questionnaire was distributed through official social media profiles on LinkedIn and Twitter.

The questionnaire did not include specific screening criteria. From August to October 2022, two hundred and forty responses (*N* = 240) were received. Three responses were omitted due to incomplete information; therefore, two hundred and thirty-seven (*N* = 237) were used for the analysis. Using MS Excel and Power BI, descriptive statistics were applied to analyse the responses obtained from the questionnaire’s categorical variables, following the guidelines provided by Spriestersbach et al. [62]. Graphical representations were used to facilitate the interpretation of findings, identify patterns, and gain insights from the collected data.

The collected data were analysed using the R statistical package (version 4.1.2). The objective was to see if there were statistical differences between the responses collected for factors impacting consumer purchases and consumer perceptions of why new alternative protein sources are introduced to the market across different age groups, educational attainment levels, and countries of residence (UK, Slovenia, and Others). The required conditions for the safe use of the parametric test were first checked to select the appropriate statistical test for each dataset (i.e., normality of each data sample involved in the comparison, independence, and the homoscedasticity of the variance) using the guidelines set by Eftimov et al. [63]. In this case, the questions were unrelated and were independent. The normality condition was checked using the Shapiro–Wilk test, while the homoscedasticity of the variance was checked by Levene’s test. A non-parametric test was selected if either condition was not satisfied. It is also important to note that all the cases dealt with paired samples.

The results of selected statistical tests for each dataset are shown separately, together with the obtained *p*-values. A *p*-value below 0.05 indicated a statistically significant difference. In cases when the Friedman test revealed a statistically significant difference, the groups responsible for that difference were determined using an all vs. all post hoc procedure (Nemenyi test). The results of this test were then used to plot the Critical Difference (CD) diagrams. In each CD diagram, groups where *p* < 0.05, i.e., no statistical difference, are connected by a bold line.

Pearson correlation coefficients were calculated to assess the linear relationship between variables in multiple-choice questions, ranging from −1 (indicating a strong negative correlation) to +1 (indicating a strong positive correlation). *P*-values have been omitted due to space constraints, as the coefficients offer substantial insight into the strength and direction of the correlations.

The CHAID (Chi-squared Automatic Interaction Detection) method was employed to analyse data on the frequency of consumption of meat alternatives across demographic segments [64,65]. The analysis was structured into multiple decision nodes, each validated by significant Chi-square values. Demographic variables such as educational attainment, gender, and age group served as independent variables in relation to the dependent variable—the frequency of consuming these alternatives. Following the validation of prerequisites for Chi-square analysis, it was determined that the method could effectively evaluate the consumption patterns of plant-based alternatives. Due to insufficient responses, insect-based alternatives failed to meet the necessary conditions for CHAID analysis, specifically for the ‘often’ and ‘very often’ categories.

### 2.2. Focus Group Design, Conduct, and Analysis

Focus groups were conducted using Breen’s [66] guidelines, with the moderator introducing the topic and setting the ground rules to ensure a respectful exchange and decorum amongst participants.

For each focus group conducted in the UK and Slovenia, purposive sampling was employed to recruit a diverse group of six individuals (*N* = 12) to gather the opinions of different age groups and educational backgrounds [67,68]. An equal distribution of male and female participants was maintained within each group for a standardised composition. These virtual discussions were conducted over Zoom from March to April 2023, lasting approximately 90 min each. Throughout each session, participants were actively encouraged to express their thoughts and perceptions in response to the prompts and questions shown in Table 1.

The focus group discussions were transcribed and analysed using MaxQDA, and template analysis was applied to identify, organise, and interpret key themes, patterns, and concepts from the data [69,70,71].

## 3. Results

### 3.1. Demographic Characteristics

Table 2 presents the demographic characteristics of the questionnaire respondents. The data shows a greater proportion of respondents in the two younger age groups (16–25 and 26–35) who had higher education degrees and identified as female. Regarding countries, responses from the UK and Slovenia are displayed, as these countries had higher response rates than other European countries. Responses from other European countries have been aggregated under the ‘Others’ category to facilitate interpretation when comparing responses with those from the UK and Slovenia. These responses were collected from Germany (14%), Belgium (6%), Sweden (5%), Italy (3%), the Netherlands (2%), less than 2% each from Spain, Greece, France, and Switzerland, and less than 0.5% from Austria and Norway.

After the questionnaire, two focus groups were conducted, each with six participants (*N* = 6). In the UK focus group, participants were evenly distributed across age groups: one from the 16–25, 26–35, 46–55, and 56–65 groups and two from the 36–45 group. Similarly, the Slovenian focus group included one participant each from the 16–25, 36–45, 46–55, and 56–65 groups and two from the 26–35 group. Both focus groups maintained an equitable male–female ratio, with three males and three females in each group.

### 3.2. Factors Affecting Consumer Food Purchases

**Figure 1:**
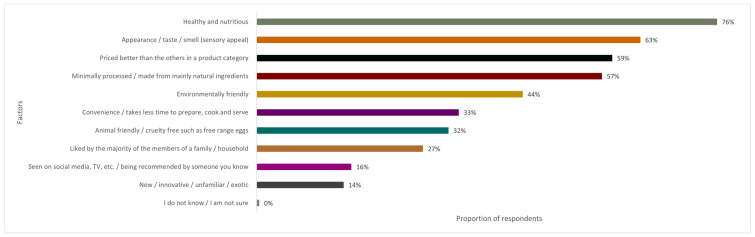
Bar graph showing factors affecting consumer purchase decisions for food items (%; *N* = 237).

Figure 1 shows the factors that influence food purchases. Respondents had the opportunity to select multiple answers relevant to their decision. The results show that ‘healthy and nutritious’ (76%) was cited as the most important factor, followed by ‘sensory appeal’ (63%), which includes appearance, taste, and smell. The third and fourth factors, ‘priced better than others’ (59%) and ‘natural or minimally processed’ (57%), were similarly selected.

‘Environmentally friendly’ (44%) ranked fifth, followed by ‘convenience’ (33%), ‘animal welfare’ (32%), and ‘liked by the majority of family members’, which was selected by 27% of the respondents. Factors indicating the influence of media and demand for new or innovative products received lower percentages at 16% and 14%, respectively, and less than 0.5% of respondents selected ‘I do not know’ or ‘I am not sure’.

Table 3 shows the analysis of these variables in relation to demographic variables. Age (*p* < 0.001; *p* < 0.05) and educational attainment levels (*p* < 0.001; *p* < 0.05) have a higher level of statistical significance, while the country of residence (*p* = 0.02; *p* < 0.05) exhibits a lower level of statistical significance. In comparison, the assessment of factors with respect to sexes (*p* = 0.06; *p* > 0.05) indicates no statistical significance.

**Figure 2:**
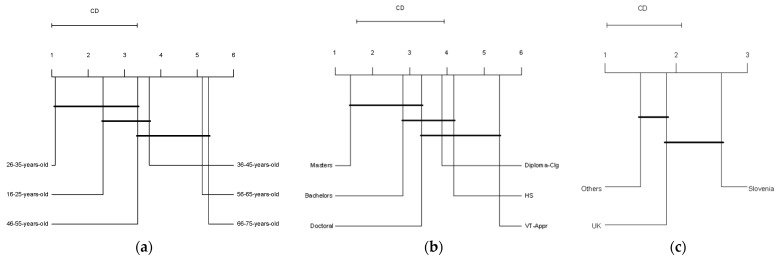
(**a**) CD diagram of the Friedman/Nemenyi test for factors affecting food purchases across different age groups. (**b**) CD diagram of the Friedman/Nemenyi test for factors affecting food purchases across different educational attainment groups. (**c**) CD diagram of the Friedman/Nemenyi test for factors affecting food purchases across the UK, Slovenia, and countries under the ‘Others’ category. (**a**–**c**) Groups are ranked from left to right based on the level of their influence on factors affecting consumer food purchases (higher rank means higher influence). Bold lines indicate no significant difference between connected groups (*p* < 0.05). The CD line at the top shows the threshold for statistical significance; differences exceeding this line are significant.

Figure 2a presents the Critical Difference (CD) diagram of the Friedman/Nemenyi test for factors impacting food purchases across different age groups. Significant differences were found between 26–35 years and 36–45, 56–65, and 66–75 years, as well as between 16–25 years and 56–65 and 66–75 years. These differences indicate a generational divide, with younger groups (16–35 years) prioritising pricing and marketing, while older groups (56–75 years) emphasise appearance and a preference for natural or minimally processed options. The analysis revealed no statistical difference in preferences between the following age groups: 26–35 years and both 16–25 and 46–55 years; 16–25 years and both 36–45 and 46–55 years; and 46–55 years and 66–75 years. These similarities suggest shared behaviours and preferences within these age groups.

Figure 2b presents a Critical Difference (CD) diagram of the Friedman/Nemenyi test for factors impacting food purchases across different educational attainment groups. These groups are denoted as HS (Secondary school/High school diploma), VT-Appr (Vocational training/Apprenticeship), Diploma-Clg (College/University/Level 3 (UK) diploma), Bachelors (bachelor’s degree), Masters (master’s degree), and Doctoral (Doctoral degree). Significant differences were identified between Masters and Diploma-Clg, Masters and HS, Masters and VT-Appr, and Bachelors and VT-Appr. These findings suggest that higher educational attainment is associated with a stronger preference for ‘healthy and nutritious’, ‘natural or minimally processed’, and ‘environmentally friendly’ foods. No statistical difference in preferences was observed between other groups. The lack of significant differences between other groups could be due to overlapping socio-economic factors that influence behaviour and preferences similarly across different educational levels.

Figure 2c presents a Critical Difference (CD) diagram of the Friedman/Nemenyi test for factors impacting food purchases across different countries. A significant difference was found between Slovenia and Others, with Slovenian respondents placing more emphasis on pricing, sensory characteristics, and the quality of food they consume. The analysis showed no statistical difference in preferences between the UK and both Slovenia and Others. This lack of difference between the UK and the other groups might be attributed to shared market access and global consumer trends.

### 3.3. Perception of New Alternative Protein Sources to Meat

**Figure 3:**
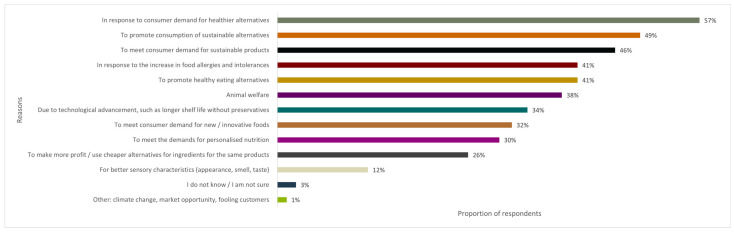
Bar chart illustrating reasons behind introducing new alternative protein sources to meat according to consumers (%; *N* = 237).

Figure 3 depicts the factors consumers consider important when asked why they think alternative protein sources to meat are being introduced to the market. Amongst the factors given, healthier alternatives in response to consumer demand (57%) had the highest percentage, followed by sustainability, which 49% saw as a means to promote sustainable alternatives, while 46% saw it as a response to consumer demand and 41% of consumers believed it was a means to promote healthier alternatives.

Another 41% of consumers attributed the demand to the increasing occurrence of food allergies and intolerances, and 30% chose the factor ‘personalised nutrition’. While the percentage for personalised nutrition is lower than that for allergies, it still signifies that consumers like those who responded to this questionnaire have seen products that offer diverse options to serve a wider range of dietary preferences.

The well-being of animals (38%) also emerged as a salient concern, and 34% of respondents believed this was due to technological advancement. A further 32% believed it was a response to consumer demand for new or innovative food products, and 26% believed that producers could make more profit using cheaper ingredients. Only 12% of consumers opted for sensory characteristics, while a small percentage of respondents expressed uncertainty (3%) or provided reasons such as climate change and marketing strategies (1%). Nevertheless, consumer awareness of food choices is gradually evolving, particularly regarding overarching concerns about general well-being, environmental impact and ethical considerations.

Table 4 shows the analysis of these factors in relation to demographic variables. Age (*p* < 0.001; *p* < 0.05) and educational attainment levels (*p* < 0.001; *p* < 0.05) were found to have a higher level of statistical significance. In comparison, the country of residence (*p* = 0.02; *p* < 0.05) and sex (*p* = 0.04; *p* < 0.05) had a lower level of statistical significance.

**Figure 4:**
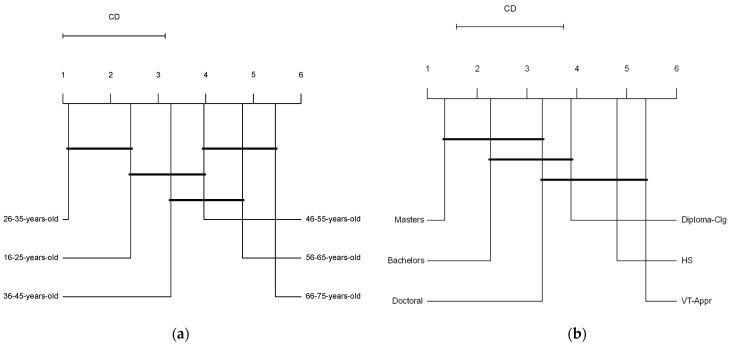
Critical Difference (CD) diagrams of the Friedman/Nemenyi test for perception of new alternative protein sources to meat across different (**a**) age groups and (**b**) educational attainment groups. (**a**,**b**) Groups are ranked from left to right based on the level of their perception of new alternative protein sources to meat (higher rank means a more positive perception). Bold lines indicate no significant difference between connected groups (*p* < 0.05). The CD line at the top shows the threshold for statistical significance; differences exceeding this line are significant.

Figure 4a presents the Critical Difference (CD) diagram of the Friedman/Nemenyi test comparing consumer perceptions of new alternative protein sources to meat across different age groups. Significant differences were found between the following pairs: 26–35 years and 36–45 years, 26–35 years and 56–65 years, 26–35 years and 66–75 years, 16–25 years and 56–65 years, and 16–25 years and 66–75 years. These findings indicate a generational divide, with younger age groups (16–35 years) focusing more on sustainable consumption and animal welfare, whilst older age groups (56–75 years) are more concerned with health and nutrition. The analysis showed no statistical difference in preferences between the following pairs of age groups: 26–35 years and 16–25 years, 26–35 years and 46–55 years, 16–25 years and 36–45 years, and 46–55 years and 66–75 years. The lack of significant differences between these pairs suggests shared behaviours and preferences within these age groups and, in cases of adjacent generational groups, it may be due to similar perceptions influenced by shared experiences or life stages.

Figure 4b presents a Critical Difference (CD) diagram of the Friedman/Nemenyi test comparing consumer perceptions of new alternative protein sources to meat across different educational attainment groups. The groups are denoted as HS (Secondary school/High school diploma), VT-Appr (Vocational training/Apprenticeship), Diploma-Clg (College/University/Level 3 (UK) diploma), Bachelors (bachelor’s degree), Masters (master’s degree), and Doctoral (Doctoral degree). Significant differences were identified between the following pairs: Masters and Diploma-Clg, Masters and HS, Masters and VT-Appr, Bachelors and VT-Appr, and Bachelors and HS. These findings suggest that higher education levels are associated with a stronger preference for health and sustainable alternatives, whilst lower education levels link the introduction of alternative proteins to the promotion of healthier options and technological advancements. No statistical difference in preferences was observed between other pairs, implying that socio-economic factors may influence behaviours and preferences similarly across different educational levels.

The correlation analysis reveals the complex links between what consumers think about the introduction of alternative protein sources to meat across different age groups. The desire for healthier options is strongly linked with all other factors (r > 0.83), highlighting that health concerns are a major reason for introducing alternative proteins. In contrast, worries about food allergies and intolerances are less connected to promoting healthy eating (r = 0.42), personalised nutrition (r = 0.37), and sustainability (r = 0.48), suggesting that these worries are seen as separate motivations. The moderate connections of sensory characteristics (r < 0.65) with other factors highlight the importance of taste and texture in making alternative proteins appealing to consumers. The results of these correlations were used to develop prompts related to motivations for alternative protein sources to meat in the focus group guide (refer to Table 1), considering key consumer concerns and motivations, particularly the strong link between health benefits and other factors. Negative correlations led to generating prompts targeting personal preferences for participants to discuss.

### 3.4. Consumer Acceptance of Different Alternative Protein Sources to Meat

When asked if respondents were familiar with the three alternative protein sources in the study, namely plant-based, CCDM, and insects, the results revealed that plant-based alternatives, such as burger patties, were recognised by 96% of participants, making them the most familiar option. Edible insects, such as cricket pasta, were chosen by 74% of the respondents, while 62% chose CCDM.

When asked if they had tried these alternative protein sources to meat, 95% of respondents reported that they had tried plant-based alternatives, showing a high level of acceptance for this option. Insect-based alternatives were tried by 23% of participants and CCDM was tried by 5% of respondents.

A breakdown of both variables (heard of and tried yet) by age, sex, educational attainment, and country of residence is shown in Table 5, serving to identify patterns indicating high or low interest in different protein alternatives amongst specific groups of respondents.

The awareness of plant-based food was consistently high across all demographics, ranging from 94% to 100%. Females showed a slightly higher awareness (99%) than males (95%). Amongst different age groups, the younger age groups (16–25 and 26–35) had the highest awareness (100%) compared to the older groups. Interestingly, educational attainment did not seem to impact awareness significantly. The UK residents had the highest awareness of plant-based food (100%), which was when compared to residents of Slovenia (94%) and other European countries (97%). This divergence can be linked to the increasing percentage of consumers in various European countries who are adopting or showing an interest in plant-based products. A study utilising Google Trends and a data analytics tool to track vegan-related queries across multiple languages placed the UK number one on the list, while Slovenia was 15th [72].

In the case of CCDM, awareness was generally lower than that for plant-based food, ranging from 31% to 81%, with females indicating slightly higher awareness (63%) than males (61%). However, when trying CCDM, males (5%) showed more interest than females (4%). Amongst different age groups, the 16–25 and 26–35 groups had the highest awareness (70% and 72%), whereas the oldest age group (66–75) had the highest proportion of respondents who had tried it (14%). A trend indicating increased awareness with higher educational attainment was observed, with higher education levels between 65% and 81% and lower educational levels below 50%. A striking observation was the similarity in the percentages obtained for awareness of CCDM amongst Slovenian residents (73%) and those in other European countries (72%), while UK residents had a comparatively lower percentage (40%). The low percentages for trying CCDM are unsurprising, considering that CCDM is unavailable in any direct consumer market in the EU.

In contrast, awareness of edible insects fell within the moderate range, with even lower percentages reported when asked about trying them. Males demonstrated higher awareness (77%) than females (73%), which also translated to trying edible insects, as a higher proportion of males (31%) compared to females (19%) had tried them. Across different age groups, the 16–25 and 26–35 groups had the highest percentage of respondents who were aware of edible insects (77% and 93%, respectively), and the 66–75 group had the highest proportion of respondents who had tried edible insects (43%). Awareness of edible insects was higher amongst individuals with a doctoral degree (92%) and a master’s degree (76%). When asked about trying edible insects, individuals with a doctoral degree (50%) had the highest percentage compared to the other groups. In terms of geographical comparison, UK residents (75%) and residents of other European countries (80%) had higher awareness of edible insects than Slovenian residents (67%). However, when trying edible insects, Slovenian residents (27%) and residents of other European countries (28%) had higher rates than residents of the UK (12%). In the analysis of alternative protein preferences, awareness of plant-based alternatives showed a moderate positive correlation with trying them (r = 0.56), suggesting that increased awareness significantly drove consumers to try these products. There was a weak positive correlation between awareness of plant-based alternatives and trying insect-based alternatives (r = 0.14), indicating some crossover in consumer interest. Awareness of edible insects also demonstrated a weak positive correlation with trying them (r = 0.40), reflecting that higher awareness somewhat increased the likelihood of trying insect-based foods. There was a weak positive correlation between awareness of edible insects and trying plant-based alternatives (r = 0.18), suggesting a slight overlap in consumer interest. Awareness of cultured meat showed a moderate positive correlation with trying insect-based alternatives (r = 0.30), indicating a shared consumer openness to novel protein sources. In contrast, the negligible correlation with trying plant-based alternatives (r = 0.01) suggested distinct consumer groups.

### 3.5. Existing Consumption Patterns for Alternative Protein Sources to Meat

Subsequently, the respondents were presented with questions to evaluate consumers’ perceptions regarding the safety of these meat alternatives and how these perceptions might impact their current consumption patterns. Using a five-point Likert scale, the respondents were asked to express their perceived level of safety for each alternative protein source. Based on their responses, the respondents were prompted to indicate the frequency of their consumption using an ordinal scale.

In response to the question, “On a scale of 1 to 5, how strongly do you believe plant-based alternatives are safe for human consumption?”, the results showed that the majority of the participants agreed with the statement (53% strongly agreed, while 32.5% agreed), indicating a high degree of certainty in the safety of plant-based alternatives. Meanwhile, 27% remained uncertain or neutral, while a small proportion of respondents disagreed (1.5% strongly disagreed, 4.5% disagreed).

When examining the data about sex, educational attainment, and age groups, the observed trends were similar to the overall findings. Key differences emerged in the oldest age group (66–75), where none of the respondents strongly disagreed and the majority either agreed (57%) or strongly agreed (14%). A similar observation was made within the educational attainment group with a doctoral degree, where no respondents strongly disagreed or disagreed and the majority either agreed (25%) or strongly agreed (50%). These agreement responses were also consistent with those observed for respondents with a master’s degree.

In terms of a country-wise comparison, it was interesting to note that none of the respondents from the UK strongly disagreed with this statement, and the UK had the highest proportion of respondents who strongly agreed (38%). In Slovenia, only a few respondents strongly disagreed (3%), and among the remaining respondents who either agreed or strongly agreed, the proportion of those who strongly agreed was lower (34%).

When asked about the consumption patterns of plant-based meat alternatives, the data showed different consumption patterns, with the highest percentage (35%) of respondents occasionally opting for these alternatives, 30% rarely consuming them, 17% consuming them frequently, and 8% showing a strong preference for consumption. In comparison, 10% avoided plant-based alternatives altogether.

**Figure 5:**
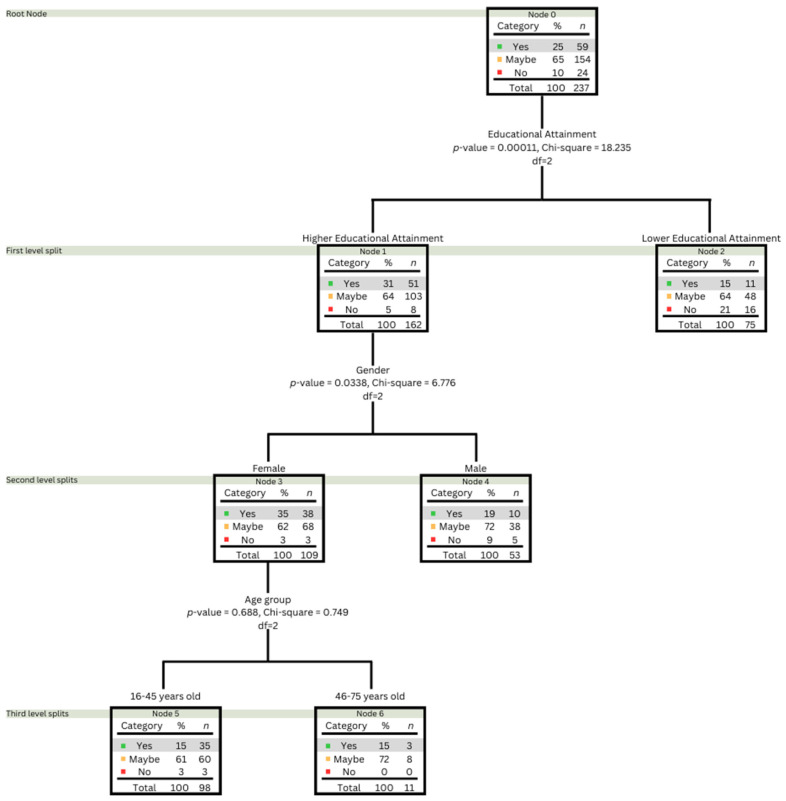
CHAID decision tree analysis of the frequency of consumption of plant-based meat alternatives by socio-demographic variables (educational attainment, gender, age group; based on Chi-square significance). The colour blocks represent the distribution of responses (‘Yes’—green, ‘Maybe’—orange, ‘No’—red) across various splits.

Figure 5 presents the findings from the CHAID analysis on the frequency of consumption of plant-based meat alternatives across socio-demographic variables. Responses to consumption frequency were categorised into three groups: Yes (includes ‘Often’ and ‘Very Often’), Maybe (includes ‘Sometimes’ and ‘Seldom/Rarely’), and No (‘Never’). The CHAID analysis identified significant variations in plant-based consumption frequency across the population. The results of the CHAID analysis showed significant differences between education and sex but not with the age group. Educational attainment emerged as the principal predictor of consumption frequency (*p* < 0.0001), leading to the initial split in the dataset.

Regarding education, the higher educational group, with 31% of respondents, had a higher acceptance of plant-based meat consumption than the lower educational group (15%). A subsequent analysis based on gender within the higher education cohort revealed a significant disparity (*p*-value = 0.0338), with females reporting higher frequencies of consumption (35%) than males (19%). Further investigation within the higher-educated female group indicated that age was not a significant factor (*p*-value = 0.688) for the 16–45 and 46–75 groups. It should be noted that the analysis was deemed valid as the main category of interest (‘Yes’) had sufficient responses to satisfy the requirements for Chi-square. A similar evaluation approach was provided by Kasza et al. [65] to understand the consumption of insect-based foods among Hungarian consumers.

The questionnaire asked respondents to rate the safety of CCDM for human consumption. The results showed that 49% of the respondents were uncertain or neutral, while 8% disagreed and 15% strongly disagreed with the statement. Conversely, 17% of the respondents agreed and 11% strongly agreed with the statement, i.e., they considered CCDM safe for consumption.

When the data were examined in relation to educational attainment and age groups, a notable trend emerged, revealing that as the age groups increased and educational attainment decreased, the perceptions of safety for CCDM tended to decrease. In terms of sex, there was only a slight deviation, with 5% more males than females perceiving CCDM as safe for consumption.

For country-wise comparison, a difference in trend was observed for residents of the UK and Slovenia. In the case of the UK, 32% of the respondents either strongly disagreed or disagreed, while the majority (59%) remained neutral, with only 8% agreeing with the safety of CCDM. In comparison, for Slovenia, 32% of the respondents remained neutral, and 16% disagreed or strongly disagreed, while a larger proportion (35%) perceived CCDM as safe, agreeing with the statement.

As discussed previously, the relatively lower familiarity, acceptance responses, and perceptions of safety regarding CCDM can be attributed to the varying regulations surrounding them in the European region. To understand where respondents stand as consumers on this issue, they were asked whether they would be open to trying CCDM once it becomes available in supermarkets, using a dichotomous question. The findings revealed that 43% responded affirmatively, while 56% responded negatively.

Respondents were then asked to rate the safety of insects for human consumption with the help of a Likert scale. The results revealed that most respondents perceived insects as safe (26% agreed and 22% strongly agreed), 35% remained uncertain or neutral, 13% disagreed, and only 5% strongly disagreed with the statement.

When the data were analysed concerning sex, the observed trends were similar to the overall findings with only a slight variation, indicating that 10% more male respondents than female respondents agreed it was safe. However, key differences emerged within the age groups. Interestingly, within the oldest age group (66–75), none of the respondents expressed strong disagreement, while none strongly concurred in the 56–65 age group. Similarly, within this age group, although no one strongly disagreed, the majority (63%) remained neutral or uncertain about the safety of edible insects.

Regarding the educational attainment group, no one with a doctoral degree strongly disagreed, and the group had the highest number of respondents who agreed with the statement (36% agreed, 28% strongly agreed). Regarding a country-wise comparison, respondents from the UK and Slovenia had perceptions similar to the overall findings in this case.

**Figure 6:**
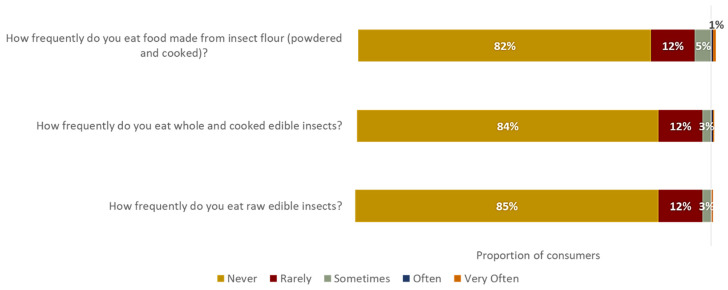
Bar chart illustrating the consumption of insect-based meat alternatives amongst consumers (%; *N* = 237).

Figure 6 presents an overview of respondents’ eating habits regarding insect-based meat alternatives in three forms: raw edible insects, whole and cooked edible insects, and insect flour in powdered and cooked form, measured on an ordinal scale.

When asked how frequently they ate raw edible insects, the results showed that most respondents (85%) never consumed them in this form. Only a small percentage (12%) chose the option that they rarely consumed raw edible insects, while even fewer respondents (3%) chose the option “sometimes”. None of the respondents reported eating raw edible insects often or very often.

Similarly, when the respondents were asked about eating whole and cooked edible insects, 84% reported never having tried them in this form. A small proportion (12%) mentioned eating whole and cooked edible insects rarely, while only 3% stated eating them sometimes. None of the respondents reported eating whole and cooked edible insects often or very often.

Regarding food made from insect flour (powdered and cooked), most respondents (82%) reported never consuming such products. A small percentage (12%) mentioned consuming food made from insect flour rarely, while 5% stated eating it sometimes. Only 1% of respondents reported eating food made from insect flour often or very often.

### 3.6. Motivations and Barriers to Consuming Different Alternative Protein Sources to Meat

Table 6 presents a content analysis of motivations driving the adoption of alternative protein sources based on focus group discussions with participants from the UK and Slovenia. Each theme and sub-theme represent key factors derived from qualitative data and encompasses various aspects that motivate participants to embrace alternative protein sources to meat. The number in the columns indicated as the UK (Freq) and Slovenia (Freq) represents the frequency of times participants from the UK and Slovenian focus groups discussed a specific sub-theme.

In the Slovenian and UK focus groups, participants articulated various reasons for embracing alternative protein sources to meat, with prominent themes centring on health considerations, environmental concerns, economic factors, curiosity, and media influence. For instance, a participant from the UK group, MR, stated, “*I think that one of the biggest issues and shortages that we are probably heading towards, as the population keeps getting bigger, is global food shortages. And I think the way we farm right now we can’t manage that population, given the higher demand. Supply and demand are not going to work long term. So, I believe that looking at alternative sources of protein is very important*”.

The lowest level of motivation pertained to preferences, which participants in both focus groups perceived more as a barrier than a driving force. They commonly expressed how preferences are hindrances rather than motivations for adopting protein alternatives. For instance, a participant from the Slovenian focus group, LC, stated, “*So maybe that’s one of the main reasons I would not really consider eating alternatives. I mean, if I have a steak available and something plant-like, there’s still a significant difference in meat’s texture, flavour, smell, and everything. So maybe when that difference is smaller or even the same, I think that the population will maybe change its mind and think that’s okay, I can have this, or I can have that, it’s the same, but until then, I think that the change is not possible*”.

The ethical concerns, encompassing both animal welfare and social welfare, received moderate attention and elicited responses from participants. In this study, most participants spoke highly of the potential positive impact of meat alternatives on animal welfare. However, in each focus group, 3–4 participants expressed concerns, particularly in the context of CCDM, about the potential effects it might have on the livelihoods of individuals engaged in livestock and animal rearing.

The media and social influence also garnered moderate attention. Interestingly, participants from both focus groups held comparable perspectives on each theme, emphasising sub-themes similarly. However, variations emerge about media influences, where the Slovenian focus group assigned less significance to target campaigns, influencers, and celebrity endorsements than their UK counterparts. Notably, four out of six participants in the Slovenian group expressed concerns that such media strategies could have a more negative impact than positive if executed “*too aggressively*” or “*forcefully*”.

In both focus groups, a prominent barrier identified by most participants was the perception that alternative options, such as plant-based meat and meat grown in a laboratory, were perceived as “*highly processed*” or formulated with “*unknown ingredients*”. The participants expressed concerns regarding the limited investigation into the potential consequences of consuming these alternatives, leading to reservations and hesitancy towards their adoption. One participant, IB from the UK focus group, highlighted the contrast between natural and artificial perceptions, stating, “*It’s probably the perception of natural versus artificial. That’s probably why you’d be more willing to eat things like ants or any sort of natural food, compared to anything that’s grown in a lab. So, as long as they can change that perception, we’re going to have a struggle where people will be hesitant to try anything that’s seen as artificial, compared to something that’s natural*”.

Regarding insects, aside from the common barriers of disgust and appearance, another significant obstacle influencing their consumption was familiarity. A participant in the Slovenian group, MM, provided insights on this matter, stating, “*It is a matter of habit and I think of our environment and culture where we grew up and what we’re used to. For example, in Australia, they eat kangaroo meat, and it’s okay for them. So, I think if we were brought up eating insects, it would not be such a dilemma*”.

In this study, participants in both focus groups expressed concern about the accessibility of alternative food options and their ability to incorporate them into their diets. They stressed the importance of learning about these alternatives and the proper preparation methods to replicate dishes they have tried elsewhere with ingredients such as insects and possible food combinations.

Affordability also emerged as a prominent barrier, given the global rise in the cost of living. For example, SG from the UK focus group stressed the importance of protein in their diet, expressing a preference for authentic chicken fillets as a high-quality, cost-efficient source of protein over alternative proteins options. NM from the Slovenian focus group echoed this sentiment, stating that the need for protein strongly influences their food choices. Should traditional meat offer a more affordable protein source than alternatives, they would prioritise it.

### 3.7. Suggestions to Overcome Identified Barriers

After discussing all the possible barriers that participants came up with, they were prompted to provide suggestions for overcoming the obstacles associated with adopting alternative protein sources to meat. In this case, the suggestions were based on options originating from plants, CCDM, and insects.

Table 7 showcases a matrix analysis comprising UK and Slovenian focus group inputs. Each sub-theme corresponds to distinct facets related to overarching themes, and the suggestions put forth by the participants offer strategies to address the identified barriers. The numerical value in parentheses accompanying each suggestion denotes its frequency of discussion amongst participants from the UK and Slovenian focus groups.

In the sensory profile domain, participants expressed concerns about the differences in texture and taste of alternative protein sources to meat. They described the alternative proteins as softer (in certain cases, crunchier), crumblier, and grainier, lacking the traditional chewiness and firmness associated with meat. In terms of flavour, particularly the processed options were either mild or had an overwhelmingly dominant flavour that felt ‘fake’ and ‘unnatural’. Participants noted that the taste and texture of these processed alternatives reminded them more of grains, vegetables, and legumes than traditional meat. Some also reported experiencing a nutty or earthy smell coming from these foods. They recommended marketing alternative protein sources as distinct or separate categories, rather than as ‘meat substitutes’, to highlight their own unique flavours and prevent direct comparisons with traditional meat and meat products. Participants also emphasised the importance of diversifying the current options on the market and offering more choices in soups, curries, and sauces to go beyond fillets and steaks. As GM from the UK focus group articulated, “*Bolognese with veggies. It’s about adding a good sauce to the pasta. I think that could create a positive experience*”.

In addition to providing dedicated resources such as recipe books and blogs to guide consumers in their culinary explorations, participants suggested introducing product samples in supermarkets to address familiarity as a barrier. As DM from the UK pointed out, “*We’re assuming everyone has tried meat-free options probably once. You’d be surprised; many people have never tried these products. If you can get people to try them at the supermarket, for example, with someone offering vegetarian sausages and asking, ‘Have you ever tried one of these?’ Many people would be surprised. I was honestly quite surprised by how tasty some of these options were myself*”.

Discussing cost and convenience, participants expressed concerns regarding the price of alternative protein sources compared to traditional meats. To address this, they put forth recommendations like implementing price reduction initiatives and bulk purchasing incentives. MT from Slovenia underlined, “*I think it’s going to be the same with insects. The price point will take precedence. The same applies to lab-grown meat. Even if its quality matches or surpasses that of animal meat, if it ends up being more affordable, I believe people would indeed be more likely to adopt it*”.

Participants drew attention to the challenges of locating these products within supermarkets. They proposed the creation of a separate section adjacent to the meat range, distinct enough to avoid any confusion. GM from the UK stated, “*I think that there were a couple of supermarkets near us that tried to mix things up a little bit and have veggie sausages with the meat sausages. But I think there were a lot of complaints because people accidentally picked up the wrong thing. You know, they saw the veggie sausages, and then there were slightly cheaper sausages mixed in the same section. They picked those up and got them home and realised that they were meat*”.

A notable knowledge gap emerged concerning ingredients and the processing methods involved, as discussed by MR and IB from the UK concerning the perception of natural versus artificial. Misconceptions about meat alternatives surfaced, prompting participants to advocate for comprehensive and easily accessible information that could disprove these myths. LC from Slovenia and RG from the UK concurred, affirming, “*Transparent information can aid in countering misinformation*”.

This discussion also gave rise to another reservation, particularly concerning CCDM. While participants initially embraced the concepts, their attitudes shifted during the discourse due to a lack of information about the long-term impact on the human body. MM from Slovenia and IB from the UK expressed reservations about consuming CCDM, classifying it as “*not the best idea*”. Participants shared this sentiment in both focus groups. As a result, half of the participants (*N* = 3) from both countries rated these alternatives at the lowest preference level for consumption.

Environmental concerns prominently featured in the participants’ discussions, inciting contemplations on the sustainability of alternative protein sources to meat. BP from Slovenia expressed, “*Understanding the environmental and animal welfare impact motivated me to adopt a vegetarian lifestyle; nothing else*”. Participants recommended showcasing initiatives that promote sustainability or revealing the resource conservation achieved through the consumption of these foods.

Within the context of media and social factors, participants acknowledged the influential role of media and societal dynamics in shaping perceptions. The UK participants suggested leveraging endorsements by celebrities and influencers to increase awareness. However, participants cautioned against campaigns that might feel forced in both focus group discussions. Exploring the integration of meat alternatives into social gatherings, inspired by existing culinary traditions like BBQ cookouts and pizza nights, emerged as another strategy.

Suggestions from UK and Slovenian participants revealed shared themes such as addressing sensory concerns, enhancing culinary variety, and recognising the importance of knowledge. Both groups emphasised the value of widening food options and the impact of social gatherings. Differences emerged regarding factors like the environment and nutrition. The UK participants prioritised transparent sustainability information and media endorsements, while Slovenian participants favoured well-crafted educational campaigns.

## 4. Discussion

This study’s strength lies in adopting a mixed-methods approach, which is well-established as a means to gain deeper insights into consumer behaviour [73]. This study found that consumers consider health-related and environmental factors as reasons for introducing alternative protein sources to meat. However, environmental concerns were ranked only fifth when evaluating the factors influencing grocery shopping amongst the respondents. During the focus group discussions, participants initially mentioned that environmental factors greatly motivate them. Nevertheless, health and nutritional reasons primarily influenced their choices to reduce meat consumption or opt for alternative dishes. This finding suggests that whilst consumers acknowledge the importance of sustainability, it may not be the sole motivation for their dietary choices.

Pricing and sensory factors were highlighted as important influencers on consumer food choices, aligning with the findings of previous studies [42,74]. As observed in the case of traditional meat consumption, taste and texture are considered paramount by consumers when it comes to alternative proteins [39]. Lang et al. [75] identified that consumers who found plant-based meat appealing in taste and texture showed positive attitudes towards it. While pricing was recognised as an important factor, participants in this study considered it less significant than sensory qualities. It should be noted, however, that this study did not account for the participants’ spending potential, which might have provided further insights into these findings.

In examining the socio-demographic variables influencing the perception and consumption of alternative protein sources, notable patterns emerged across age groups, educational attainment levels, and genders. Younger respondents, particularly those between 16 and 35, demonstrated a higher propensity to try alternative protein sources to meat, suggesting an increased openness to innovative foods. This trend contrasted with older age groups, who showed less inclination towards such alternatives, potentially due to more ingrained dietary habits or scepticism towards non-traditional food sources.

Educational attainment plays a crucial role, as individuals with higher educational credentials perceived these proteins as safer and were more likely to consume them. Specifically, women with higher educational levels expressed a strong preference for plant-based meat alternatives, highlighting a gender-specific trend where educated females are leading in adopting plant-based diets [31,76].

Gender differences were evident in the perception of unconventional proteins such as edible insects, with men displaying a greater inclination towards considering insects safe and being more willing to consume them than women. This finding aligns with findings from Kasza et al. [65], indicating that men typically exhibit lower levels of neophobia, positioning them as early adopters of innovative products. Kulma et al. [77] highlighted that young men tend to be more experimental and open to trying insects, an observation consistent with the findings of this study regarding gender dynamics in entomophagy. Minimally processed or fresh ingredients were also found to be important factors based on the findings of the questionnaire and the focus group discussions. In considering this factor, plant-based alternatives emerged as an interesting point of discussion, boasting the highest acceptance rate in terms of safety and frequency of consumption compared to other meat alternatives. Despite this widespread acceptance, however, plant-based alternatives still lag behind traditional meat products in terms of consumption levels, a trend consistent with findings from other studies [78,79].

In addition to differences in dietary preferences, one key reason participants from the focus groups in both countries gave that accounted for this discrepancy was the possibility of chemical contamination in these foods [80] and concerns about undisclosed chemicals in the ingredient list of plant-based protein alternatives [81]. Most participants agreed that they would instead opt for simple vegetable-based meals rather than vegetables processed to look like meat if they want to avoid traditional meat altogether.

In the case of edible insects, despite their known benefits, notable barriers to their consumption associated with disgust and neophobia are prevalent [82]. Although participants in the focus group did not explicitly express disgust towards eating insects, there was evident hesitation in their responses, corroborating previous research findings [83,84,85] and the questionnaire findings. Coinciding with the outcomes of Meyer-Rochow and Hakko [86], only a small fraction of respondents indicated any degree of insect consumption in the questionnaire.

Drawing insights from the focus group responses in both the UK and Slovenia, participants suggested that it is more likely for edible insects to be more widely accepted if their presentation is altered within food products or they are integrated into familiar dishes that consumers are more accustomed to in a particular region [87], as demonstrated in studies with powdered grasshopper [88] and termite [89]. This notion also resonates with the discussion by Meyer-Rochow and Hakko [86], who proposed that processing insects into flour or pastes increases the likelihood of consumer acceptance. Further, the study of Ribeiro et al. [32], performed in Portugal and Sweden, indicates that acceptance of products incorporating processed insects was higher than acceptance of whole unprocessed insects. For a category such as insects to create a positive impression, cooperation amongst government bodies, scientists, and businesses is crucial to ensure that products are available to consumers and that information about food safety and consumption of insects is accessible. Piha et al. [57] found greater consumer acceptance of insects in Northern Europe than in Central Europe, citing the influence of institutional frameworks and cultural and dietary differences.

Specifically examining how consumers perceive CCDM, a lab-grown meat alternative, the study found that overall perceptions of CCDM are neutral, with half of the consumers who believe CCDM is safe expressing willingness to try it. Factors such as animal welfare and concerns about environmental sustainability were also primary motivators for consumers who expressed this interest, a finding that again aligns with previous research, such as that discussed by Hocquette et al. [90], which also highlights consumer interest in cultured meat, primarily driven by ethical and environmental concerns, particularly animal welfare. Liu et al. [91] investigated the impact of socio-demographic factors on consumer awareness of meat consumption issues, finding that women, older respondents, and individuals with higher levels of education tend to be more receptive to ethical and environmental concerns associated with meat consumption. These factors also shape perceptions of meat alternatives like CCDM, indicating that attitudes towards conventional meat strongly influence perceptions of meat alternatives. Consumers with critical views of conventional meat are more likely to view CCDM favourably.

In the questionnaire, 5% of respondents reported having tried CCDM, an unexpected finding possibly attributable to their personal experiences outside the European region and their interpretation of what qualifies as CCDM. Some individuals might have travelled internationally to regions where cultured meat is not restricted and had the opportunity to try it there. Additionally, there could be misunderstandings or misperceptions about what constitutes cultured meat [90], with respondents possibly mistaking other meat products for cultured meat due to unclear labelling or misunderstanding [92].

During the focus group discussions, while CCDM was initially welcomed as a beneficial innovation, some participants also debated its potential downsides, such as perceptions of being unnatural and potentially harmful to the human body, lack of knowledge about the production process and impact on the environment, inconsistencies in legislations, as well as concerns about its impact on jobs in traditional animal agriculture, a sentiment consistent with the observations of previous research [93,94,95]. These concerns also caused hesitation amongst the participants regarding the introduction of CCDM in the European market. If CCDM were to be introduced to European consumers, stakeholders would need to address the ambiguities and gaps surrounding CCDM and engage consumers to address their questions and concerns, to ensure successful market adoption [96,97].

Amongst the participants, there was a unanimous consensus that information about the ingredients in processed meat alternatives should be accessible and available for consumers to educate themselves about these products and gain a clear understanding of them. This finding echoes the sentiments discussed by Fanelli et al. [98], emphasising the importance of ingredient transparency in building consumer trust in food safety.

This study also revealed consensus amongst participants regarding marketing these products as a distinct category rather than being grouped with traditional meat products. This finding agrees with other published findings that consumers were more inclined to try plant-based meat alternatives when labelled as plant-based rather than meat alternatives [99]. An interesting observation emerged concerning supermarkets. All participants in the focus groups expressed an interest in alternative protein products, particularly plant-based options, but discussed the challenge of locating these products on market shelves, especially when time is limited. This observation aligns with the perspective presented by Gravely and Fraser [36], who emphasised that while supermarkets expand the availability of plant-based choices, they employ rudimentary marketing strategies for these products within the store. With current sales reaching USD 11.3 billion at a 6.7% year-on-year (YOY) growth in 2021, and projected sales set to exceed USD 22.5 billion at a 7.2% compound annual growth rate (CAGR) by 2032 [100], this presents a substantial opportunity for supermarkets to address the growing consumer demand.

In addition to the benefits of utilising the influence of product names, descriptions, and packaging [99,101], focus group participants offered three simple solutions to improve the accessibility and visibility of these alternative protein sources to meat: strategic product placement on shelves, dedicated shelf sections, and the presence of on-site sampling representatives. A study by Ejlerskov et al. [102] highlights differences in purchasing behaviour at checkout points of supermarkets among consumers. The study also discusses how variation in socio-demographic variables, particularly age groups, influences grocery purchasing. These observations are consistent with the results presented in this study. This is an interesting outcome because it indicates that on-site marketing strategies might not prove as effective if insights from one consumer group are generalised for the whole population. It is necessary to consider the perspectives of various consumer groups if stakeholders aim to promote the adoption of alternative protein sources across the population rather than targeting a specific consumer segment.

This study’s findings also highlighted the role of media and social gatherings in shaping consumer attitudes, since nearly all participants in the focus groups tried alternative proteins through personal connections. The influence of socio-cultural factors has been widely discussed in the literature [38,103]. Social media has had positive effects in promoting plant-based foods [104] and meat substitutes [105]. Thus, leveraging endorsements or strategies that resonate with societal perceptions could boost the adoption of meat alternatives.

An interesting observation was the variation in responses of the focus group participants in the UK and Slovenia. For instance, the UK participants favoured a more aggressive marketing approach, using mainstream media and celebrity endorsements to increase awareness and acceptance, whereas the Slovenian participants advocated for more subtle and educational marketing strategies. According to the participants, these suggestions were based on their experiences with how campaigns differ in their respective countries, which demonstrates that despite similarities in factors corresponding to the barriers and motivation, strategies to encourage the adoption of alternative protein sources should be tailored to fit the cultural and societal context of each country. Although the consumption of specific protein alternatives, like insects, remains unconventional in the Western setting, insights drawn from the focus group discussions suggest that providing information in a tactful and non-coercive manner could be advantageous. This concept resonates with the conclusions of Gahukar [106], who emphasised the establishment of educational programs, robust entrepreneurial endeavours, academic research, and government support to promote the recognition of insects as a significant commodity in society.

### Limitations and Future Directions

We acknowledge certain limitations of our study. The relatively small sample size of participants for both the questionnaire and the focus group affects the generalisability of the findings. In the questionnaire, the representativeness of the results is potentially compromised by the uneven distribution of respondents across countries, as a predominant number of responses from certain regions may introduce geographical bias. Another limitation emerges from the socio-demographic representation, particularly regarding the questionnaire respondents’ age groups and educational attainment levels. Despite efforts to ensure that the focus group participants represented diverse age groups, educational backgrounds, and professional levels, the interview guide used for the focus group discussions may have disproportionately reflected the viewpoints of specific consumer groups, thus potentially introducing bias into the responses obtained.

A limitation of this study is the predominance of participants from the UK and Slovenia. Whilst their contributions offer valuable insights, it is important to recognise the potential constraints in generalising the findings to broader geographical regions or different European cultural landscapes, which include varied dietary preferences and traditions. Variations in levels of consumer awareness and acceptance of alternative protein sources can significantly influence perceptions and are likely distinct across different European regions. Consequently, applying these results beyond their immediate social environment would present challenges in predicting outcomes in other locations or for people with contrasting societal dynamics.

Future researchers are encouraged to pursue a more heterogeneous sample of participants, encompassing different countries and ensuring equal representation of various consumer segments. This approach would expand the scope and boundaries for a better understanding of consumer perceptions towards alternative protein sources to meat and provide a foundation for drawing broader overarching conclusions and implications on a global scale.

## 5. Conclusions

In this study, combining a questionnaire with focus group discussions gathered detailed insights into motivations and barriers concerning alternative protein sources to meat and facilitated participant-driven suggestions that addressed the identified barriers. In addition, this study drew comparisons from respondents from countries with varying legislative systems within the European region. This study has been centred on understanding how consumers perceive alternative protein sources such as plant-based, CCDM, and insect-based foods. Positive attitudes often arise from health considerations, environmental awareness, and ethical values, while obstacles like texture, safety concerns, and a sense of unnaturalness have been identified. It is important to note that consumer preferences are notably influenced by socio-cultural factors, the media, and the individuals within their immediate circles. A crucial element that fosters consumer acceptance is providing clear and comprehensive information, complemented by imaginative and innovative strategies to enhance the visibility of such products within supermarket premises. These facets should be effectively leveraged when devising consumer-centric strategies within strategic marketing. It is essential to identify the appropriate target audience and tailor strategies accordingly for all knowledge-based and educational initiatives. These components may collectively play an instrumental role in encouraging consumers to adopt alternative protein sources instead of meat.

## Figures and Tables

**Table 1:** Focus group discussion segments and prompts.

Segments	Prompts
Introduction	Self-introduction and dietary habits
	Usual protein sources in the diet
	Understanding of alternative proteins
Motivation	Experience with meat substitutes
	Reasons for interest
	Positive experiences
	Understanding the benefits of alternative protein sources to meat
	Personal preferences
Barriers	Negative experiences
	Concerns about physiochemical properties such as taste and texture
	Concerns about plant alternatives
	Concerns about insects
	Concerns about cell-culture-derived meat
	Other concerns related to availability and affordability
	Personal preferences
Suggestions	Opinion on existing strategies/campaigns
	Suggestions for industry and government
	Suggestions that would work for other consumers
	Impact of addressing barriers
Wrap-up	Final thoughts from participants
	Questions regarding alternative protein sources to meat

**Table 2:** Demographic characteristics of questionnaire respondents (%; *N* = 237).

Demographics	% of Sample
Sex	
Female	67%
Male	33%
Age group (in years)	
16–25	24%
26–35	41%
36–45	13%
46–55	14%
56–65	5%
66–75	3%
Educational attainment	
Doctoral degree	15%
Master’s degree	34%
Bachelor’s degree	19%
College/University/Level 3 (UK) diploma	13%
Vocational training/Apprenticeship	7%
Secondary school/High school diploma	12%
Country of residence	
UK	33%
Slovenia	32%
Others	35%

**Table 3:** Summary of statistical analysis results on factors affecting consumer food purchases.

Variables	Methods	*p*-Values
Age	Friedman	<0.001 (9.61 × 10^−8^)
Country of residence	Friedman	0.02
Education	Friedman	<0.001 (2.73 × 10^−5^)
Gender	Wilcoxon Signed-Rank	0.06

Statistical significance is determined at α = 0.05 level.

**Table 4:** Summary of statistical analysis results on the perception of new alternative protein sources to meat.

Variables	Methods	*p*-Values
Age	Friedman	<0.001 (6.56 × 10^−9^)
Country of residence	Repeated-Measures ANOVA	0.02
Education	Friedman	<0.001 (3.55 × 10^−8^)
Gender	Wilcoxon Signed-Rank	0.04

Statistical significance is determined at α = 0.05 level.

**Table 5:** Variables ‘Heard of’ and ‘Tried yet’ for different alternative protein sources in terms of respondents across age, sex, educational attainment, and country of residence (%; *N* = 237).

	Plant-Based Meat Alternative		Cell-Culture-Derived Meat		Edible Insects and Insect-Based Food	
Demographics	% (Heard of)	% (Tried yet)	% (Heard of)	% (Tried yet)	% (Heard of)	% (Tried yet)
**Sex**						
Female	99%	97%	63%	4%	73%	19%
Male	95%	95%	61%	5%	77%	31%
**Age group (in years)**						
16–25	100%	100%	70%	7%	63%	19%
26–35	97%	96%	72%	4%	77%	26%
36–45	97%	93%	67%	7%	93%	23%
46–55	97%	97%	31%	0%	72%	22%
56–65	100%	92%	31%	8%	69%	15%
66–75	86%	86%	57%	14%	86%	43%
**Educational attainment**						
Doctoral degree	92%	92%	81%	6%	92%	50%
Master’s degree	99%	95%	76%	4%	76%	31%
Bachelor’s degree	98%	98%	65%	9%	74%	17%
College/University/Level 3 (UK) diploma	100%	100%	47%	3%	73%	7%
Vocational training/Apprenticeship	100%	100%	6%	0%	69%	6%
Secondary school/High school diploma	97%	97%	48%	7%	55%	3%
**Country of residence**						
Slovenia	94%	91%	73%	6%	67%	27%
UK	100%	100%	40%	6%	75%	12%
Others	97%	97%	72%	4%	80%	28%

**Table 6:** Content analysis of motivations for adopting alternative protein sources to meat (f; *N* = 12).

Themes	Sub-Themes	UK (Freq)	Slovenia (Freq)
Environmental	Sustainability	6	7
	Climate change awareness	6	4
	Carbon footprinting	4	1
	Increasing population impact	2	1
Health and nutritional concerns	Health benefits	8	6
	Nutritional benefits	6	7
	Digestive health	4	4
	Dietary restrictions	3	4
Economic factors	Affordability	5	6
	Availability	6	4
Convenience	Time constraints	3	4
	Easy meal preparation	4	5
Curiosity	Trying new foods	5	5
	Creativity in food preparation	4	4
	Exploring diversity in food	4	4
Ethical	Animal welfare	4	5
	Social welfare	3	4
Social influence	Family	4	3
	Friends	3	3
Media influence	Advertisement impact	6	4
	Target campaigns	6	3
	Celebrity endorsements	6	2
Preference	Taste	2	3
	Familiarity with ingredients	3	3
	Variety	2	3
	Texture	3	2

**Table 7:** Matrix analysis of suggestions to overcome barriers to adopting alternative protein sources to meat in the UK and Slovenia (n; *N* = 12).

Themes	Sub-Themes	Participant Suggestions (UK/Slovenia)
Sensory profile	Concerns about texture	Market alternative protein products as distinct options, not just as comparisons to traditional meats (UK: 6, Slovenia: 6)
	Concerns about taste	Introduce a broader range of options in soups, curries, and sauces rather than focusing solely on fillets and steaks (UK: 4, Slovenia: 5)
Awareness	Familiarity with varieties	Address unfamiliarity with different varieties by offering samples in supermarkets for consumers to try (UK: 5, Slovenia: 2)
	Familiarity with cuisine	Provide targeted recipe books and blogs featuring different dishes that can be prepared using alternative protein sources to meat (UK: 3, Slovenia: 3)
Cost and convenience	Cost compared to traditional meat	Implement price reduction initiatives and bulk purchasing incentives (UK: 4, Slovenia: 5)
	Difficulty finding products in supermarkets	Ensure products have a separate section close to the meat range but distinct to avoid confusion (UK: 2, Slovenia: 2)
Knowledge gap	Chemical vs. natural ingredients and processing	Offer transparent information about ingredients and processing methods (UK: 5, Slovenia: 4)
	Misconceptions about alternatives	Provide accessible, comprehensive information for consumers (UK: 3, Slovenia: 3)
	Nutritional value	Emphasise health and nutritional benefits over traditional meat (UK: 3, Slovenia: 4)
	Environmental impact awareness	Share supported sustainable initiatives or provide transparency on resource conservation due to alternative protein consumption (UK: 3, Slovenia: 2)
Media and social factors	Mainstream media influence	Leverage endorsements by celebrities and influencers (UK: 3, Slovenia: 0)
	Negative effect of campaigns	Design educational campaigns with care to avoid seeming forced (UK: 4, Slovenia: 6)
	Social gatherings	Explore incorporating alternative protein sources to meat into social gatherings inspired by BBQ cookouts, pizza nights, and hotpot outings (UK: 4, Slovenia: 4)
	Influence of cultural norms	Promote a diverse food culture and present alternative protein sources to meat as an experience tied to different cultures (UK: 4, Slovenia: 2)

## Data Availability

All related data and methods are presented in this paper. Additional inquiries should be addressed to the corresponding author.

## Supplementary Materials

The following supporting information can be downloaded at https://www.mdpi.com/article/10.3390/foods13111627/s1, Table S1: Questionnaire (core components).
