# Supplementary material for: Is Sustainable Consumption a Sufficient Motivator for Consumers to Adopt Meat Alternatives? A Consumer Perspective on Plant-Based, Cell-Culture-Derived, and Insect-Based Alternatives
Source: Foods. 2024 May 23;13(11):1627. doi: 10.3390/foods13111627 (PMC11171576; doi:10.3390/foods13111627)
Supplement: Supplementary file 1 [file foods-13-01627-s001.zip › foods-2959758-supplementary.pdf]

Appendix A – Questionnaire (core components)

1 Which factors are important to you when buying groceries? Choose as many as you like.

A Healthy and nutritious

B Minimally processed / made from mainly natural ingredients

C Environmentally friendly

D Animal friendly / cruelty free such as free-range eggs

E Appearance / taste / smell (sensory appeal)

F Convenience / takes less time to prepare, cook and serve

G Priced better than the others in a product category

H Liked by the majority of the members of a family / household

I New / innovative / unfamiliar / exotic

J Seen on social media, TV, etc. / being recommended by someone you know

K I do not know / I am not sure

L Other: ______________________________________

2 *New alternative foods are defined as alternatives to foods prepared or produced by traditional methods. There are currently many foods on the markets that are available as alternatives to traditional meat and dairy products.*

As a consumer, why do you think alternative foods are being introduced to the market? Choose as many as you like.

A In response to consumer demand for healthier alternatives

B To promote healthy eating alternatives

C To meet the demands for personalised nutrition

D In response to the increase in food allergies and intolerances

E To meet consumer demand for new / innovative foods

F For better sensory characteristics (appearance, smell, taste)

G To promote consumption of sustainable alternatives

H To meet consumer demand for sustainable products

I Animal welfare

J Due to technological advancement, such as longer shelf life without preservatives

4 Which alternative foods from the following list have you tried? Choose as many as you like.

A Plant-based alternatives

B Lab-grown meat alternatives

C Insect-based alternatives

5 How strongly do you believe plant-based alternatives are safe for human consumption?

1	2	3	4	5
Strongly disagree		Not sure/ Neutral		Strongly agree

6 How frequently do you eat plant-based alternatives?

1	2	3	4	5
Never		Some-times		Very Often

7 How strongly do you believe lab-grown meat alternatives are safe for human consumption?

1	2	3	4	5
Strongly disagree		Not sure/ Neutral		Strongly agree

8 Lab-grown meat alternatives are not yet widely available to buy. If that changes, would you include lab-grown meat alternatives in your diet?

Yes	No

9 On a scale of 1 to 5, how strongly do you believe insects are safe for human consumption?

1	2	3	4	5
Strongly disagree		Not sure/ Neutral		Strongly agree

10 On a scale of 1 to 5, how frequently do you eat raw edible insects?

1	2	3	4	5
Never		Some-times		Very Often

11 On a scale of 1 to 5, how frequently do you eat whole and cooked edible insects?

1	2	3	4	5
Never		Some-times		Very Often

12 On a scale of 1 to 5, how frequently do you eat food made from insect flour (powdered and cooked)?

1	2	3	4	5

K	To make more profit / use cheaper alternatives for ingredients for the same products	Never	Some-times	Very Often
L	I do not know / I am not sure			
M	Other: ____________________________________			
3	Which alternative foods from the following list have you heard of? Choose as many as you like.			
A	Plant-based alternatives e.g., burger patties			
B	Lab-grown meat / cell culture-derived meat			
C	Edible insects / insects as food e.g., cricket pasta			
